# ^23^Na MRI quantification of sodium content in porcine eyes after immersion in saltwater and freshwater *en route* to time in water estimation

**DOI:** 10.1186/s41747-025-00605-x

**Published:** 2025-07-09

**Authors:** Tobias Lindner, Adrian Konstantin Luyken, Chris Lappe, Oliver Stachs, Thoralf Niendorf, Matthias Lütgens, Stefan Polei, Brigitte Vollmar, Andreas Buettner, Sönke Langner, Marc-André Weber, Ebba Beller

**Affiliations:** 1 Core Facility Multimodal Small Animal Imaging, University Medical Center Rostock, Rostock, Germany; 2 Institute of Diagnostic and Interventional Radiology, Pediatric Radiology and Neuroradiology, University Medical Center Rostock, Rostock, Germany; 3 Department of Ophthalmology, University Medical Center Rostock, Rostock, Germany; 4 Berlin Ultrahigh Field Facility (B.U.F.F.), Max Delbrück Center for Molecular Medicine in the Helmholtz Association, Berlin, Germany; 5 Rudolf-Zenker-Institute for Experimental Surgery, University Medical Center, Rostock, Germany; 6 Institute of Legal Medicine, University Medical Center, Rostock, Germany

**Keywords:** Drowning, Forensic medicine, Lens (crystalline), Post-mortem examination, Vitreous body

## Abstract

**Background:**

Differentiation between saltwater and freshwater immersion as well as estimating the corpse’s time in water can be challenging. We aimed to establish and examine the feasibility of a novel approach based on sodium magnetic resonance imaging (^23^Na MRI) of the eye to facilitate noninvasive sodium quantification.

**Methods:**

Enucleated porcine eyes were immersed in NaCl 0.9%, NaCl 3.0%, NaCl 5.85%, distilled water (DW) or lake water (LW) at different time intervals, followed by ^23^Na 7-T MRI sodium quantification.

**Results:**

After 6 h of immersion, a significant difference in vitreous body (VB) sodium concentration was found for NaCl 5.85% *versus* DW or LW (*p* ≤ 0.019). After 24 and 48 h of immersion, a significant difference in VB sodium concentration was found for NaCl 5.85% *versus* DW, LW, NaCl 3.0% or NaCl 0.9%, as well as for NaCl 3.0% *versus* DW, LW or NaCl 0.9% (*p* ≤ 0.001). After 24 h of immersion, lens sodium concentration showed a significant difference for NaCl 5.85% *versus* DW, LW, NaCl 3.0% or NaCl 0.9% (*p* ≤ 0.009); after 48 h of immersion, for NaCl 5.85% *versus* DW, LW, NaCl 3.0% or NaCl 0.9% (*p* ≤ 0.001), as well as for NaCl 3.0% *versus* DW, LW or NaCl 0.9% (*p* ≤ 0.007). For VB, sodium concentration changes over immersion time, and exponential curves were fitted to the data.

**Conclusion:**

Using ^23^Na MRI in *ex vivo* porcine eyes with different immersion times in various saltwater concentrations and freshwater equivalents allowed noninvasive quantification of VB and lens sodium concentrations.

**Relevance statement:**

Although not a substitute for autopsy, ^23^Na MRI assessment of VB and lens sodium concentrations may provide biochemical support in suspected drowning, especially in cases where an internal examination of the body is not authorized or where objections to autopsy are upheld.

**Key Points:**

- Postmortem porcine eyes with different immersion times in saltwater and freshwater.
- Noninvasive quantification of vitreous body and lens sodium concentrations with ^23^Na MRI.
- Exponential time course of vitreous body sodium concentration in saltwater and freshwater.

**Graphical Abstract:**

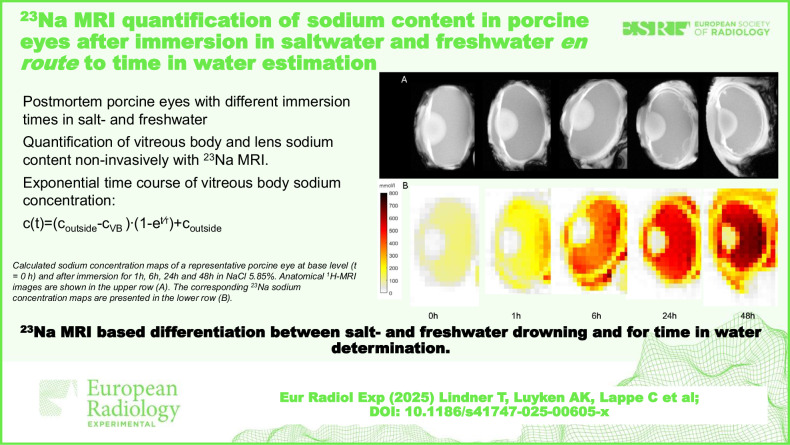

## Background

Drowning, defined as a “process of experiencing respiratory impairment from submersion/immersion in liquid” by the World Health Organization [1], is one of the leading causes of unintentional death worldwide [2]. Death by drowning commonly entails a challenging diagnostic task, resulting in a comprehensive medicolegal investigation being performed [3, 4]. Multidisciplinary efforts are often required to provide context of the actual reason for and mode of death in drowning cases, since not all corpses found in water perished from drowning. The corpse’s time in the water continues to be one of the main concerns, which aids in determining the time of death and is therefore crucial to any medicolegal investigation [5]. For this purpose, diverse methods have been developed [5–9]; however, an accurate time in water estimation is still difficult to obtain.

The vitreous humor has been thoroughly studied as a biofluid for forensic purposes via chemical, biochemical, toxicological, and metabolomic approaches to address not only the cause but also the time since death. The postmortem modifications in vitreous potassium concentration have probably been the most studied biological parameter to determine the time since death [10]. Some previous studies using bovine eyes as an animal model have established that vitreous electrolyte levels, including sodium, can change in seawater immersion, assisting in estimating the time of immersion of bodies found in water [11, 12]. Previous studies also demonstrated that the vitreous body (VB) sodium level alone was useful to differentiate between saltwater and freshwater immersion [13, 14]. However, in all previous studies, the samples of vitreous humor had to be collected via syringe aspiration. The use of imaging for quantification of VB sodium content and for differentiation between saltwater and freshwater immersion is unexplored so far.

Sodium magnetic resonance imaging (^23^Na MRI) constitutes a valuable approach for *in vivo* measurement of tissue Na^+^ concentrations [15, 16]. Increased availability and sensitivity of high-field MRI scanners, advanced MR scanner hardware and improved imaging methodology tailored for ^23^Na MRI have facilitated assessment of tissue sodium content in a broad spectrum of applications [17–26]. The feasibility of submillimeter spatial resolution ^23^Na MRI of the human eye *in vivo* facilitated clear distinctions of sodium concentration between the lens, vitreous and aqueous humor [27].

Recognizing this opportunity, this study examines the feasibility of ^23^Na MRI for the quantification of VB sodium concentrations in postmortem porcine eyes *en route* to a novel approach for differentiation between saltwater and freshwater immersion and for time in water estimation.

## Methods

### *Ex vivo* porcine eye model and immersion solution

Enucleated porcine eyes (German Landrace Swine lat. sus scrofa domesticus; age: 3 to 6 months; weight: 40 to 90 kg; ≤ 36 h after sacrificing animals) were obtained from the Research Institute for Farm Animal Biology (FBN). Following enucleation, the eyes were incubated at 8 °C in a chamber containing different immersion solutions for a fixed period of time (1 h, 3 h, 6 h, 24 h, or 48 h). For incubation, the study design involved six groups (each with *n* = 3 porcine eyes for each time point): baseline with no immersion, immersion in NaCl 0.9%, NaCl 3.0%, NaCl 5.85%, distilled water or lake water (Sildemower See). On average, ocean water salinity equals approximately 3.5% [28]. However, the salt content at the water surface differs from sea to sea and depends on tide, flow and weather conditions, *e.g*., the salinity of an intertidal beach can vary over a large range between 24 and up to 220 g/L [29]. NaCl 3.0% was therefore used as a standardized, commercially available seawater equivalent, similar to average ocean water salinity and NaCl 0.9% and NaCl 5.85% to account for variations. Lake water typically has about 1% of the salt content of ocean water, but is also subject to fluctuations and only reflects the sodium concentrations of the local area [30]. Therefore, distilled water was additionally used as a standardized solution to represent an immersion fluid with low sodium concentration.

### Ethical statement

The pigs were slaughtered according to the standard procedure for the food chain and then supplied to the commercial food market. The slaughterhouse is approved in accordance with the directives of the European Union and certified by QS (Quality and Security GmbH). It has been a member of the Animal Welfare Initiative (Gesellschaft zur Förderung des Tierwohls in der Nutztierhaltung mbH) since 2016. According to the Mecklenburg-Western Pomerania State Office for Agriculture, Food Safety and Fisheries (LALLF), Rostock, Germany, this study is not an animal experiment within the meaning of Section 7 (2) of the Animal Welfare Act, and, consequently, does not require approval (LALLF file number: 7221.3-18057_25_1).

### MRI

**Fig. 1:**
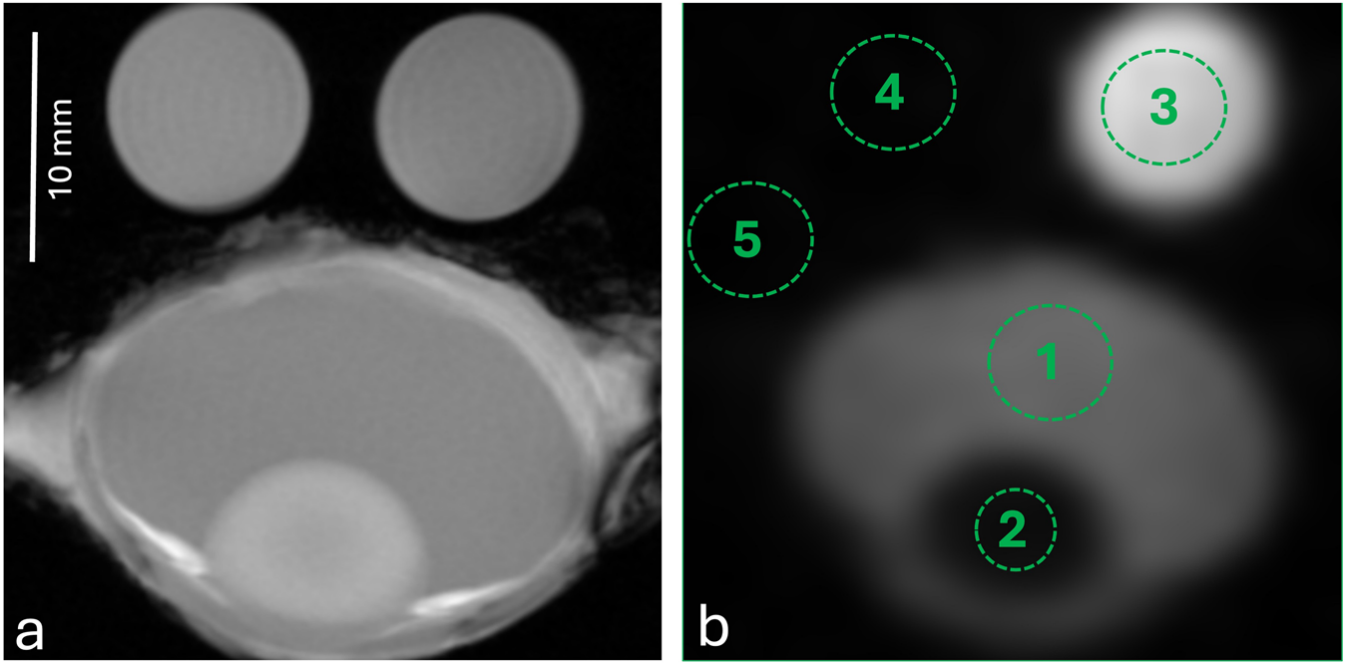
Representative example of ^1^H-MRI (**a**) and ^23^Na-MRI (**b**) of an enucleated porcine eye after incubation for 48 h in distilled water. Measurements of mean signal intensity were performed with ROIs placed in the vitreous humor (1), lens (2), NaCl 0.9% external standard (3), and distilled water reference (4, not visible in ^23^Na-MRI). Noise was obtained from the standard deviation of the background (5). For correct ROI placement, anatomical ^1^H images (**a**) were used to identify the corresponding anatomical structures. MRI, Magnetic resonance imaging; ROI, Region of interest

MRI was performed on a 7-T scanner (BioSpec 70/30, maximum gradient amplitude 200 mT/m, Bruker BioSpin MRI GmbH) operating at 300.33 MHz for ^1^H MRI and 79.44 MHz for ^23^Na MRI. A dual ^1^H/^23^Na radiofrequency volume resonator (T20118V3, Bruker, diameter = 40 mm) was used for transmission and signal reception. ^23^Na MRI of the eyes was performed using a two-dimensional low flip angle gradient echo technique: repetition time = 40 ms; echo time = 2.35 ms; excitation flip angle = 90°; matrix size = 32 × 32; field of view = 40 × 40 mm^2^; slice thickness = 5 mm; in-plane spatial resolution = 1.25 × 1.25 mm^2^; number of excitations 2,000; receiver bandwidth 25 kHz; total acquisition time = 42 min. Corresponding ^1^H MRI of the eyes was performed for anatomical imaging using a two-dimensional low flip angle gradient echo technique: repetition time = 50 ms; echo time = 2.3 ms; flip angle = 40°; matrix size = 256 × 256; field of view = 40 × 40 mm^2^; slice thickness = 5 mm; in-plane spatial resolution = 0.156 × 0.156 mm^2^; number of excitations 6; total acquisition time = 95 s. MRI was performed at room temperature within the bore of the scanner (19–21 °C). For sodium quantification, an external NaCl standard was deployed. For this purpose, two vials with different solutions (NaCl 0.9% with a sodium concentration of 154 mmol/L, as well as distilled water with a conductivity of 3.38 μS/cm, which corresponds to approximately 0.0272 mmol/L) were used as reference solutions (Fig. 1a). The eyeball and phantoms were positioned at the center of the RF coil along the *x*-, *y*-, and *z*-axis.

### Data analysis

Quantitative image analysis was performed blinded to the experimental setting. Region-of-interest-based measurements were drawn on the ^23^Na images in the VB, lens and both phantoms. Additionally, a region-of-interest was placed in the image background outside the eye and reference solutions for signal-to-noise estimation. For correct placement of the regions of interest, the anatomical ^1^H images were used for the identification of the described anatomical structures (Fig. 1). The signal intensities in the areas of the phantoms with known sodium concentrations were used as control points to assign a sodium concentration to the signal intensity in the vitreous and lens of the eye using linear fitting. Sodium concentration maps were calculated on a pixel-by-pixel basis using an in-house MATLAB script based on the calculations of Grist et al [31].

### Statistical analysis

Sodium concentrations of VB and lens were determined as median ± range, as well as their 95% confidence intervals (CIs) were calculated. To compare the differences of VB and lens sodium concentrations between different immersion solutions at different time points, one-way ANOVA, followed by Bonferroni corrections for multiple comparisons, was used. Normality of data was tested according to the Shapiro–Wilk test. *p*-values ≤ 0.05 were considered significant. All statistical analysis was performed using GraphPad Prism 10.

## Results

### VB and lens sodium concentration depending on the immersion solution

**Fig. 2:**
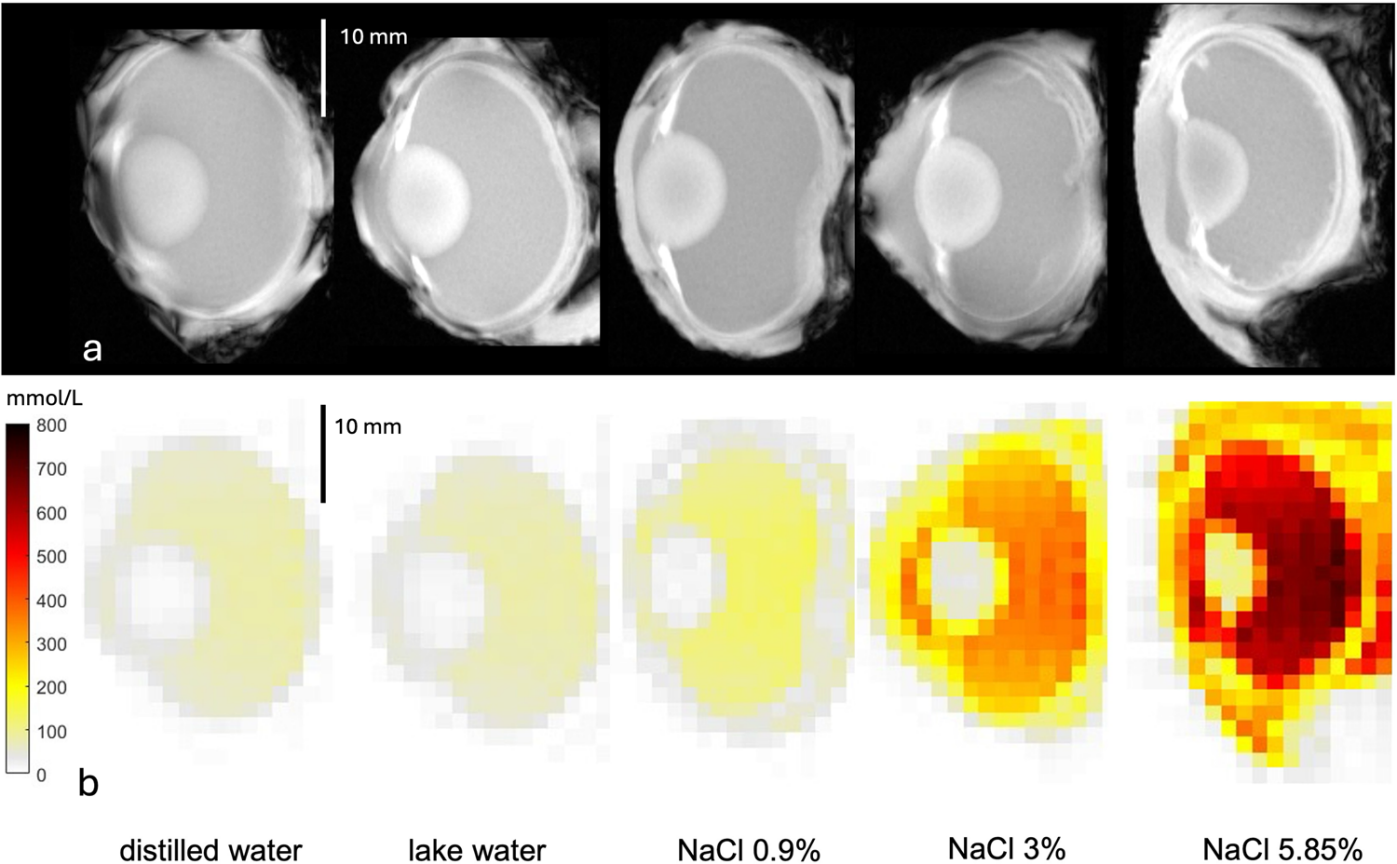
Sodium concentration maps visualize the differences in the sodium concentration in the vitreous after 48 h of immersion in different solutions. Representative example of five porcine eyes immersed for 48 h in distilled water, lake water, NaCl 0.9%, NaCl 3.0% or NaCl 5.85% with their ^1^H-MRI anatomical images shown in the upper row (**a**) and ^23^Na sodium concentration maps shown in the lower row (**b**). MRI, Magnetic resonance imaging

**Fig. 3:**
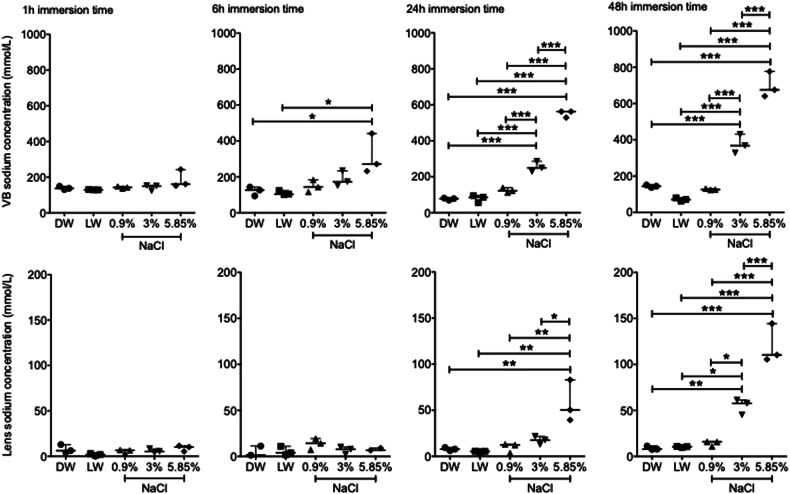
Differences between postmortem vitreous body (VB; upper row) and lens sodium concentrations (lower row) depending on the immersion solutions (distilled water (DW), lake water (LW), NaCl 0.9%, NaCl 3.0% and NaCl 5.85%), and time interval of immersion (1, 6, 24, and 48 h); *p* < 0.05 is labeled as *, *p* < 0.01 as **, and *p* < 0.001 as ***

No statistical difference was observed after 1 h of immersion time for VB or lens sodium concentration between different immersion solutions, including distilled water, lake water, NaCl 0.9%, NaCl 3.0% or NaCl 5.85%. After 6 h of immersion, a significant difference for VB sodium concentration was found between distilled water and NaCl 5.85% (*p* = 0.019) as well as lake water and NaCl 5.85% (*p* = 0.013) but no significant difference regarding lens sodium concentration (*p* > 0.05). After 24 h, a significant difference for VB sodium concentration was measured between NaCl 5.85% and distilled water, lake water, NaCl 3.0% or NaCl 0.9%, as well as NaCl 3.0% and distilled water, lake water or 0.9% (*p* ≤ 0.001). Similar results were found for VB sodium concentration after 48 h of immersion time (Fig. 2). Lens sodium concentration showed a significant difference between NaCl 5.85% and distilled water (*p* = 0.002), lake water (*p* = 0.001), NaCl 0.9% (*p* = 0.003) or NaCl 3.0% (*p* = 0.009) after 24 h of immersion and between NaCl 5.85% and distilled water, lake water, NaCl 3.0% or NaCl 0.9% (*p* ≤ 0.001, each), as well as NaCl 3.0% and distilled water (*p* = 0.003), lake water (*p* = 0.004) or NaCl 0.9% (*p* = 0.007) after 48 h of immersion. No significant difference was observed for VB and lens sodium concentration between distilled water, lake water and NaCl 0.9% after 1, 6, 24 or 48 h of immersion (*p* > 0.05) (Fig. 3).

### VB and lens sodium concentration over immersion time

**Fig. 4:**
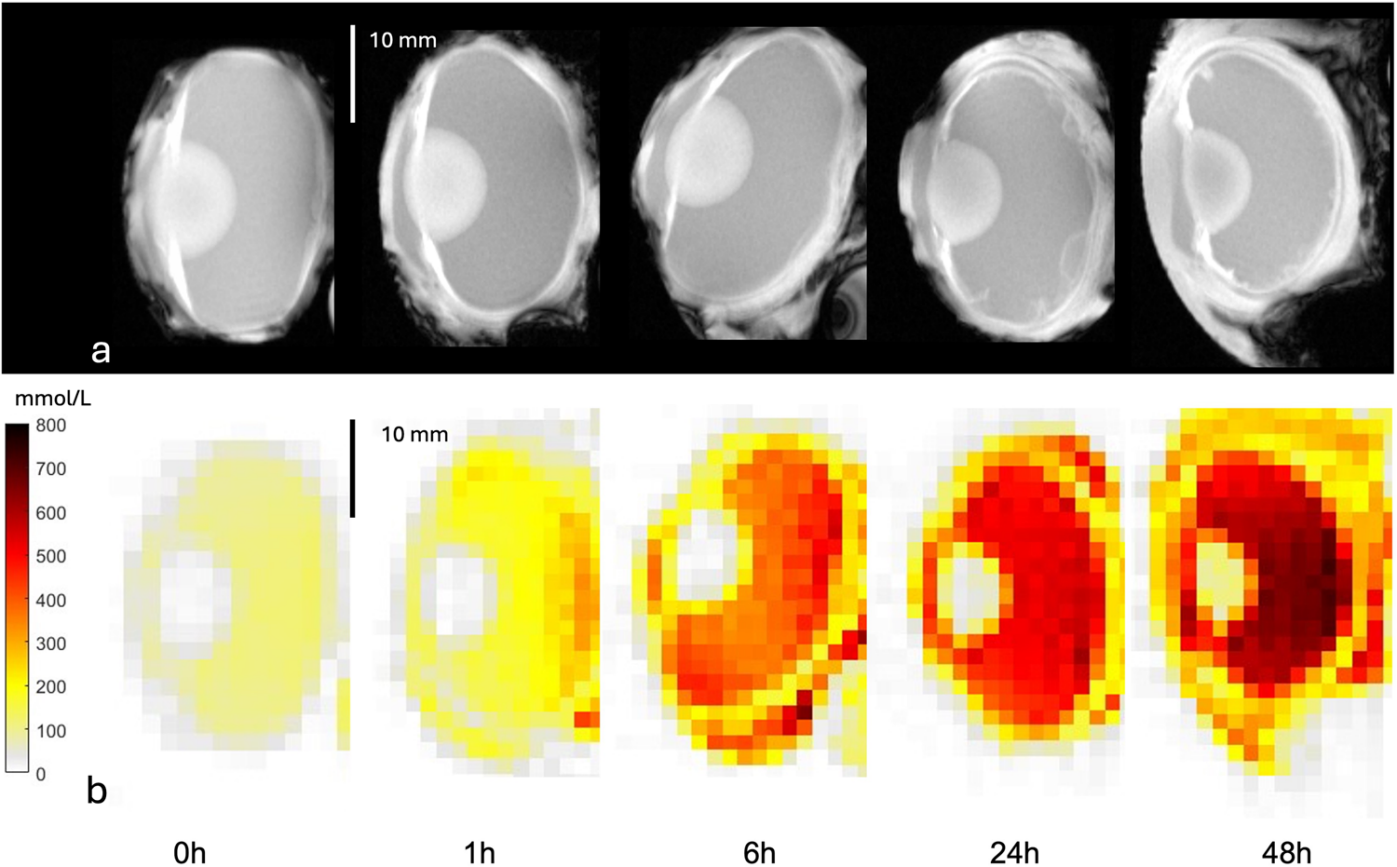
Calculated sodium concentration maps of a representative porcine eye at base level (t = 0) and after immersion for 1, 6, 24, and 48 h in NaCl 5.85%. Anatomical ^1^H-MRI images are shown in the upper row (**a**). The corresponding ^23^Na sodium concentration maps are presented in the lower row (**b**)

**Fig. 5:**
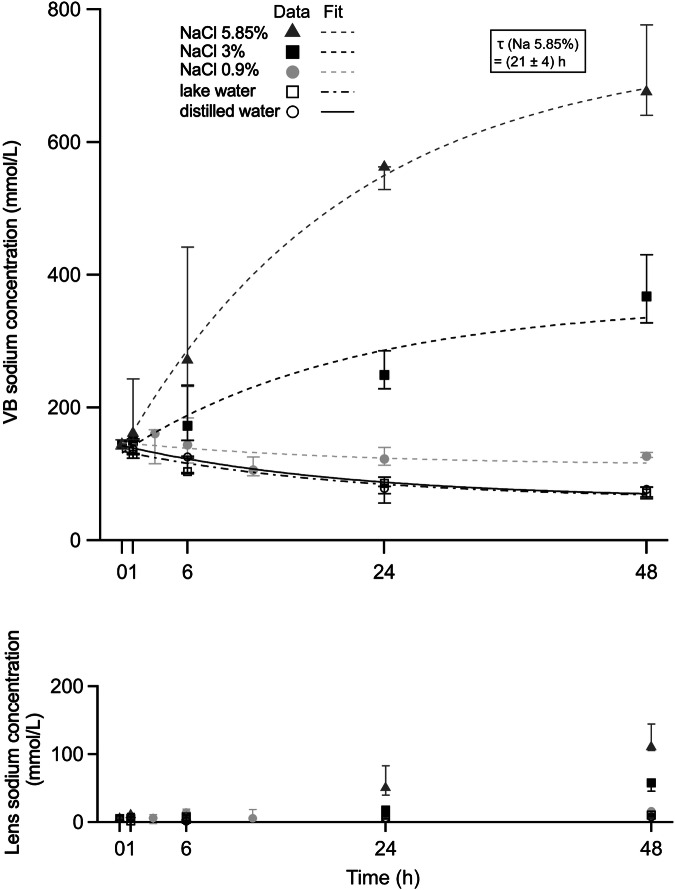
VB (upper row) and lens sodium concentration (lower row) at base level and at different time points of immersion in NaCl 0.9%, NaCl 3.0%, NaCl 5.85%, distilled water, or lake water. The exponential curves were fitted to the data according to Eq. 1 (see text). VB, Vitreous body

As shown in Fig. 5, VB sodium concentration over immersion time seems to follow an exponential time course. Therefore, a simple phenomenological approach was used to describe the time dependent change of the sodium concentration in the vitreous:

1 $${{\rm{c}}}({{\rm{t}}})=({{{\rm{c}}}_{{\mbox{outside}}}}-{{{\rm{c}}}_{{\mbox{VB}}}}) \cdot (1-{{{\rm{e}}}}^{-{{\rm{t}}}{{ / }}{{\rm{\tau }}}})+{{{\rm{c}}}_{{\mbox{VB}}}}$$

where c_outside_ represents the sodium concentration of the immersion solution, c_VB_ represents the initial sodium concentration within the vitreous body and the time constant τ incorporating the properties of the underlying diffusion process. The most significant changes of VB sodium concentration within 48 h were observed for the NaCl 5.85% immersion solution (Fig. 4). Hence, data of all other VB sodium concentration changes were fitted with fixed τ taken from the NaCl 5.85% immersion solution fit $(\tau \,=\,(21\,\pm \,4)h)$, assuming normal diffusion, which does not depend on concentration differences. The fitting coefficients obtained for each immersion solution are provided in Table 1. Figure 5 displays the fitted exponential curves showing an increase of median sodium concentration in the vitreous body over 48 h when immersed in NaCl 3.0% and NaCl 5.85%, and a decrease when immersed in NaCl 0.9%, distilled water and lake water. Assessment of the lens sodium concentration revealed no clear exponential increase or decrease and only small changes in median sodium concentration within 48 h compared to vitreous body sodium concentration (Fig. 5).

**Table 1:** Sodium concentrations in the lens and vitreous body

Immersion solution	Time (h)	Sodium concentration (mmol/L)		Fit coefficients (mmol/L)	
		Lens	VB	c_outside_	c_VB_
Control group	0	5.2 (3.1–9.3), (95% CI, -2.0 to 13.7)	144.5 (137.5–151.2), (95% CI, 137.5–151.2)		
NaCl 0.9%	1	6.6 (3.3–7.1), (95% CI, 0.6–10.9)	142.9 (136.5–149.1), (95% CI, 127.2–158.5)	113 ± 13	147 ± 9
	3	6.1 (-2.1 to -11.2), (95% CI, -11.7 to -21.8)	160.9 (115.5–166.5), (95% CI, 78.1–217.2)		
	6	14.2 (7.4–19.4), (95% CI, -1.2 to 28.6)	143.7 (116.5–184.2), (95% CI, 63.5–232.8)		
	12	5.7 (3.9–18.5), (95% CI, -10.5 to -29.2)	105.7 (97.0–125.3), (95% CI, 73.3–145.3)		
	24	12.4 (3.7–13.2), (95% CI, -3.3 to -22.8)	122.3 (113.0–139.7), (95% CI, 91.4–158.6)		
	48	15.9 (11.1–16.1), (95% CI, 7.3–21.4)	126.5 (124.5–132.1), (95% CI, 117.9–137.5)		
NaCl 3%	1	5.2 (3.7–8.2), (95% CI, 0.01–11.4)	150.1 (123.6–152.9), (95% CI, 123.6–152.9)	358 ± 28	132 ± 20
	6	7.7 (2.4–10.1), (95% CI, -3.1 to 16.6)	172.7 (150.6–233.6), (95% CI, 150.6–233.6)		
	24	17.6 (12.8–21.6), (95% CI, 6.5–28.2)	249.2 (228.3–285.7), (95% CI, 228.3–285.7)		
	48	57.6 (45.2–61.2), (95% CI, 33.8–75.5)	367.5 (327.7–430.4), (95% CI, 327.7–430.4)		
NaCl 5.85%	1	10.2 (5.3–11.6), (95% CI, 0.9–17.2)	161.3 (152.0–243.2), (95% CI, 60.8–310.2)	741 ± 36	135 ± 12
	6	6.7 (-0.6 to -9.0), (95% CI, -7.4 to -17.5)	271.3 (231.9–441.4), (95% CI, 38.3–591.5)		
	24	50.3 (39.4–83.0), (95% CI, 1.2–113.9)	562.3 (528.6–562.6), (95% CI, 502.7–599.6)		
	48	110.3 (105.5–144.3), (95% CI, 67.5–172.6)	675.5 (640.3–776.4), (95% CI, 522.0–872.9)		
Distilled water	1	6.1 (4.1–12.9), (95% CI, -3.7 to -19.1)	137.3 (130.2–149.5), (95% CI, 114.8–163.2)	62 ± 7	143 ± 5
	6	1.4 (-0.6 to -11.2), (95% CI, -11.7 to -19.7)	125.4 (100.0–126.5), (95% CI, 59.9–183.0)		
	24	6.2 (7.8–9.5), (95% CI, 3.8–11.9)	78.1 (70.0–81.3), (95% CI, 62.0–91.0)		
	48	7.9 (7.6–11.3), (95% CI, 3.8–14.1)	76.5 (65.1–80.0), (95% CI, 54.5–93.2)		
Lake water	1	1.8 (0.9–3.0), (95% CI, 0.9–3.0)	129.1 (128.9–130.5), (95% CI, 127.4–131.6)	61 ± 9	134 ± 6
	6	3.9 (1.9–10.9), (95% CI, 1.9–10.9)	103.6 (102.2–124.2), (95% CI, 79.2–140.7)		
	24	5.3 (5.1–5.4), (95% CI, 5.1–5.4)	85.7 (56.1–95.3), (95% CI, 28.3–129.8)		
	48	10.5 (9.8–10.8), (95% CI, 9.8–10.8)	71.2 (62.5–80.2), (95% CI, 49.2–93.4)		

Median (range) of sodium concentrations in the lens and vitreous body of enucleated pig eyes at base level and after different time points of incubation in NaCl 0.9%, 3%, 5.85%, distilled water or lake water. Data of vitreous body sodium concentration were fitted according to Eq. 1 to determine c_outside_ and c_VB_

*c* Concentration, *CI* Confidence interval, *h* Hour, *VB* Vitreous body

## Discussion

Our study demonstrates the feasibility of ^23^Na MRI-based quantification of VB and lens sodium concentrations in *ex vivo* porcine eyes. Our results show major differences in vitreous and lens sodium concentrations obtained for immersion in saltwater and freshwater equivalents. We also detected changes in the vitreous and lens sodium concentrations as a function of the immersion time. These findings provide an important foundation *en route* to differentiation between saltwater and freshwater immersion and for time in water estimation of bodies found in water using ^23^Na MRI.

State-of-the-art imaging is of increasing interest for forensic and legal medicine in postmortem investigations [32]. Although widely used in clinical medicine, routine application of MRI in forensic medicine is limited so far due to longer acquisition times and higher costs, when compared to computed tomography, resulting in missed vital opportunities. MRI excels in longitudinal studies with exquisite anatomical detail. The use of postmortem, whole-body proton MRI has been emerging as a powerful forensic tool with good performance regarding determination of time and cause of death as well as depiction of traumatic findings in corpses [33–36]. Recent technology allows, even on standard clinical, proton-only MRI scanners, the additional implementation, of non-proton MRI, which is based on the detection of the nuclei of atoms in the body other than ^1^H (X-nuclei), such as sodium (^23^Na), phosphorus (^31^P), chlorine (^35^Cl), potassium (^39^K), deuterium (^2^H), oxygen (^17^O), lithium (^7^Li), and fluorine (^19^F) [37]. In forensic medicine, especially ^23^Na quantification, in addition to conventional proton MRI, might be an intriguing unexplored frontier because sodium ions are one of the most important electrolytes in the human body and play a critical role in osmoregulation and cell physiology. Furthermore, disorders of plasma sodium are the most common electrolyte disturbances in clinical medicine, and severe hypo- and hypernatremia are associated with considerable morbidity and mortality [38].

While an autopsy affords external and internal access to the target anatomy for the investigation of drowning, postmortem MRI can be useful for nondestructive documentation of these findings [1]. However, it has to be noted, due to limited access to MRI scanners for medicolegal institutes in practice, the classic fluid sampling by means of biopsy and subsequent analysis of the fluid is more easily applied, which includes flame photometry [39, 40], ion selective electrode analysis [11, 13, 41], or atomic absorption spectrophotometry [42, 43] and benchtop nuclear magnetic resonance spectroscopy [44]. Nevertheless, all of these applications are limited due to invasive tissue acquisition and sample measurements, unlike ^23^Na MRI, which can be implemented on clinical whole-body MRI scanners and not only allows noninvasive determination of the sodium concentration in the vitreous body, but also has the potential to measure sodium concentration maps of other body parts [45]. For example, ^23^Na MRI of ischemic stroke has been proposed as a noninvasive tool for estimation of time after onset of tissue death [46, 47].

In our study, the median sodium concentration in the porcine vitreous was 144.5 mmol/L at base level, which is similar to sodium concentrations derived from biochemical and histological assessment of postmortem changes to the eyes of domestic swine (about 146 mmol/L) [48]. However, the median lens sodium concentration derived from our study was 5.2 mmol/L, whereas a previous study measured a mean sodium concentration of 23.6 mmol/kg in the swine lens using atomic absorption spectrophotometry [49]. These differences could be due to our measurements being centered in the lens nucleus to avoid overlap with the vitreous and the lens nucleus being less hydrated than the lens cortex [50, 51].

Our study showed that swine vitreous and lens sodium levels, when immersed in saltwater equivalents (NaCl 3.0% and 5.85%), appear to follow an exponential function. Previous studies, using bovine eyeballs as surrogates, compared the changes in bovine vitreous sodium levels when immersed in saltwater with a sodium concentration of 480 mmol/L [12] or 551 mmol/L [11]. These studies showed that bovine vitreous sodium levels were steady [12] or only showed a change per hour of 5.7 mmol/L in the first hour and increased significantly after 1 h, with an hourly increase of 16.3 mmol/L [11]. A similar experimental setting was chosen in our study when immersing in NaCl 3.0%, which has a sodium concentration of 513 mmol/L. However, we measured a change of the vitreous sodium level of 5.6 mmol/L in the first hour and of 4.5 to 5.1 mmol/L per hour after 1 h. This could be due to a lower incubation temperature of 8 °C in our study compared to 19 °C [11] and between 15 and 25 °C [12], which results in slower diffusion activity [52]. Another reason could be due to using a different specimen model with different features regarding the vitreous, *e.g*., bovine vitreous humor having a higher diffusion coefficient compared to porcine vitreous humor [53].

The diagnosis of drowning and differentiating the drowning medium (freshwater and saltwater) constitutes a challenge in forensic medicine and is largely based on the autopsy results, which exclude other causes of death and are interpreted in relation to the circumstances of death [1, 9, 54]. Also, assessment of time in water of the dead body can be challenging since changes of decomposition generally occur more slowly in water than on land and are affected, *inter alia*, by salinity, temperature, marine life, bacterial composition, currents, absence of flies and fly larvae, and contact with underwater objects. In addition, decomposition in saltwater is slower than in freshwater, as bacterial growth is delayed due to the higher salt content [1]. Similar to our study, previous studies have shown that vitreous sodium levels can be used to differentiate the drowning medium since they are increased in cases of saltwater drownings and decreased in freshwater drownings [13, 14, 55]. Nevertheless, there is no consensus regarding whether this results from hemodilution or hemoconcentration following inhalation/swallowing of water or if it is due to postmortem diffusion between the vitreous and surrounding water [12, 13, 55]. However, Zilg et al found that long immersion time was associated with lower sodium levels in freshwater drownings and additionally found no decrease in sodium levels in cases with a very short immersion time, suggesting that there is no effect on vitreous sodium concentration of hemodilution due to water inhalation [55]. Even if serum electrolyte imbalances occur, it is unlikely that they would show in the vitreous after circulatory arrest, as it also takes some time for the sodium blood levels to equilibrate with the vitreous levels [56]. In addition to that, the effect of hemodilution due to water inhalation can quite certainly be ruled out in the lens, since it is usually an avascular organ [57]. Therefore, measurements of postmortem VB and also lens sodium concentrations might provide a potentially useful ancillary test in differentiating saltwater and freshwater immersion, as well as the period between entry into the water and recovery of the dead body [58].

This study involved limitations that warrant acknowledgment. Despite the small number of samples per group, this study showed that ^23^Na MRI is sensitive to changes of sodium concentration in the vitreous and eye lens and that it is worth consideration and warrants further research. Future studies should involve a larger number of samples and ^23^Na MRI investigations at different temperatures and time points to evaluate the applicability of the method to daily forensic practice. Furthermore, the limits in time range for this method should be noted, since the sodium concentration approaches an equilibrium with time, due to its nonlinear nature, at which retrospective fit may be very inaccurate. This challenge has been previously worked out for stroke and ^23^Na MRI by others [47, 59]. However, ^23^Na MRI of the postmortem VB for time in water estimation may work very well within a 24 to 48-h period, as our data suggest. Another limitation is that we obtained our data for porcine eyes and not for human eyes. However, literature data shows that the porcine eye closely resembles the human eye [60, 61], so we can suppose that our results can be considered predictive of postmortem ^23^Na MRI of the human eye. Our study and previous similar studies [11, 12] used enucleated eyes, therefore having large areas of exposed sclera immersed, compared to most probably less than half of the surface in real water corpses. Of course, this might lead to overestimation of sodium permeability in total, making this experimental setting only very partially indicative. To perform a more realistic analysis of the immersion process, the presence of an eye socket and eyelids could provide a more impermeable interface between the immersion solution and the vitreous or lens, rendering a more delayed change in sodium concentration. General technical challenges of ^23^Na MRI include low spatial resolutions, as well as lower gyromagnetic ratio and significantly lower biological concentrations of sodium relative to hydrogen [62]. They also include partial volume effects due to tissue mixing in relatively low-resolution voxels and curved surfaces. These might especially affect the sodium measurements of the eye lens due to its anatomical features and overall low biological concentration of sodium in comparison to the vitreous.

In conclusion, ^23^Na MRI has the potential to evaluate changes of sodium concentration in the VB and lens of dead bodies found in water and therefore could be included in a postmortem multidisciplinary scenario in which, integrating data from various medical branches, a differentiation between saltwater and freshwater immersion as well as reliable estimation of time in water could be obtained. Although not a substitute for autopsy, ^23^Na MRI assessment of vitreous and lens sodium concentrations may provide biochemical support in suspected drowning, especially in cases where an internal examination of the body is not authorized or where objections to autopsy are upheld. In this light, our preliminary study provides an important contribution *en route* to ^23^Na MRI-based differentiation between saltwater and freshwater immersion and for time in water determination.

## Data Availability

The datasets used and analyzed during this study are available from the corresponding author on reasonable request.

## Abbreviations

MRI: Magnetic resonance imaging
VB: Vitreous body
